# Analyzing M-CSF dependent monocyte/macrophage differentiation: Expression modes and meta-modes derived from an independent component analysis

**DOI:** 10.1186/1471-2105-9-100

**Published:** 2008-02-17

**Authors:** Dominik Lutter, Peter Ugocsai, Margot Grandl, Evelyn Orso, Fabian Theis, Elmar W Lang, Gerd Schmitz

**Affiliations:** 1 Institute for Clinical Chemistry and Laboratory Medicine, University of Regensburg, D-93042 Regensburg, Germany; 2 Institute of Biophysics, University of Regensburg, D-93040 Regensburg, Germany

## Abstract

**Background:**

The analysis of high-throughput gene expression data sets derived from microarray experiments still is a field of extensive investigation. Although new approaches and algorithms are published continuously, mostly conventional methods like hierarchical clustering algorithms or variance analysis tools are used. Here we take a closer look at independent component analysis (ICA) which is already discussed widely as a new analysis approach. However, deep exploration of its applicability and relevance to concrete biological problems is still missing. In this study, we investigate the relevance of ICA in gaining new insights into well characterized regulatory mechanisms of M-CSF dependent macrophage differentiation.

**Results:**

Statistically independent gene expression modes (GEM) were extracted from observed gene expression signatures (GES) through ICA of different microarray experiments. From each GEM we deduced a group of genes, henceforth called *sub-mode*. These *sub-modes *were further analyzed with different database query and literature mining tools and then combined to form so called *meta-modes*. With them we performed a knowledge-based pathway analysis and reconstructed a well known signal cascade.

**Conclusion:**

We show that ICA is an appropriate tool to uncover underlying biological mechanisms from microarray data. Most of the well known pathways of M-CSF dependent monocyte to macrophage differentiation can be identified by this unsupervised microarray data analysis. Moreover, recent research results like the involvement of proliferation associated cellular mechanisms during macrophage differentiation can be corroborated.

## Background

Since microarray technology has become one of the most popular approaches in the field of gene expression analysis, numerous statistical methods have been used to provide insights into the biological mechanisms of gene expression regulation. The high dimension of expression data and the complexity of the regulatory mechanisms leading to transcriptional networks still forces statisticians and bioinformaticians to examine available methods and to develop new sophisticated approaches. However, there are already appropriate methods using different approaches to examine the underlying biological mechanisms determining the gene expression signatures and profiles measured by microarray experiments. Supervised methods using prior knowledge like *Gene Set Enrichment Analysis *(GSEA) deliver useful results under certain conditions. But there is still a lack of reliable data needed for non-classical analysis. Widely used unsupervised approaches, like hierarchical clustering and *k*-means clustering, use correlations or other distance or similarity measures to identify genes with similar behavior under similar conditions. But these methods are not able to represent more complex structures and interdependencies in the regulatory machinery.

In contrast to the algorithms mentioned above, independent component analysis (ICA) explores higher-order statistics to decompose observed gene expression signatures (GES), which form the rows of the input data matrix, into statistically independent gene expression modes (GEM), which form the rows of matrix **S **according to the data model **X**^*T *^= **AS**. ICA solves blind source separation (BSS) problems, where it is known that the observed data set represents a linear superposition of underlying independent source signals. But it can more generally be considered a matrix decomposition technique which extracts informative features from multivariate data sets like, for example, biomedical signals like EEG (Electroencephalography) [1,2], MEG (Magnetoencephalography) [3] and fMRI (functional magnetic resonance imaging) [4-6] recordings. ICA can also be considered a projective subspace technique appropriate for noise reduction [7,8], or artifact removal [9,10] if generated from independent sources.

In this work we will concentrate on the linear case, in which each single microarray GES is considered a linear superposition of unknown statistically independent GEM. To decompose these mixtures into statistically independent components, ICA algorithms like FastICA or JADE have been used. Typically, these GEMs can be interpreted as being characteristic of ongoing, largely independent biological regulatory processes. The philosophy behind can be expressed as: *co-expression means co-regulation*. But the complexity of gene regulation and the various interactions of cellular processes demands a new interpretation of our ICA-derived components. In the following we use these extracted GEMs to generate *sub-modes*, which may provide biological pathway information. The genes contained in these pathway-associated *sub-modes *can be regarded as more or less self-contained parts of larger regulatory networks, which can be represented by combining these *sub-modes *into *meta-modes *according to the functional role of the associated genes.

Here we used M-CSF dependent in vitro differentiation of human monocytes to macrophages as a model process to demonstrate that ICA is a useful tool to support and extend knowledge-based strategies and to identify complex regulatory networks or novel regulatory candidate genes.

The major known pathways associated to M-CSF receptor dependent signaling [11-13] include expansion of the role of the MAP-kinase pathway [14,15] and Jun/Fos, Jak/Stat and PI-3 kinase [16-18] dependent signal transduction. Up-regulation of immune-regulatory components involved in innate immunity response (e.g. MHC), specific (e.g. Fc) [19-21] and nonspecific (CRP, complement, galectins) [22-26] opsonin receptors as well as charge and motif pattern recognition receptors (e.g. SR-family, LRP, Siglecs etc.) [27-30], is characteristic for monocyte/macrophage differentiation. Beyond this, an increase of membrane biogenesis, vesicular trafficking and metabolic pathways including amino acids, glucose, fatty acids and sterols, as well as increased activity of lysosomal hydrolases that enhance phagocytotic function [31,32], autophagy [33] and recycling is triggered through M-CSF signaling as a hallmark of innate immunity [34]. These mechanisms are tightly coupled to changes in cytokine/chemokine response [35] and red/ox signaling (NOS e.g. NADPH-Oxidase, Glutathione, Thioredoxin, Selenoproteins) that drive chemotaxis migration, inflammation (e.g. Nf*κ*B), apoptosis (eg. Caspases, TP53, Nf*κ*B, ceramide) and survival [36-42].

## Results and Discussion

M-CSF dependent monocyte to macrophage differentiation involves the activation and regulation of many different cellular pathways. In this study we used several microarray experiments and combined them to a data set, which we analyzed using the JADE algorithm. The extracted GEMs were labeled from 1 to 14, according to decreasing energy. Note that the extracted GEMs show positive as well as negative components. They are partitioned into a *sub-mode *containing the negative signals only, denoted by *i*.1, and a corresponding *sub-mode *of the positive signals, denoted by *i*.2, respectively. These *sub-modes *were then combined into so-called *meta-modes *according to the following super categories deduced from the MeSH-filter used: *Apoptosis, signal transduction, cell cycle *and *regulatory sequences*, see table. Sub-classification and mapping to distinct pathways was then performed with the extracted *sub-modes *using the BiblioSphere MeSH- and GeneOntology-filter tools. Note that our method not only takes into account that one gene can be part of more than one pathway, but also that one pathway can be involved in more than one cellular event. This cannot be achieved with classical clustering tools.

### Signal Transduction

**Figure 1:**
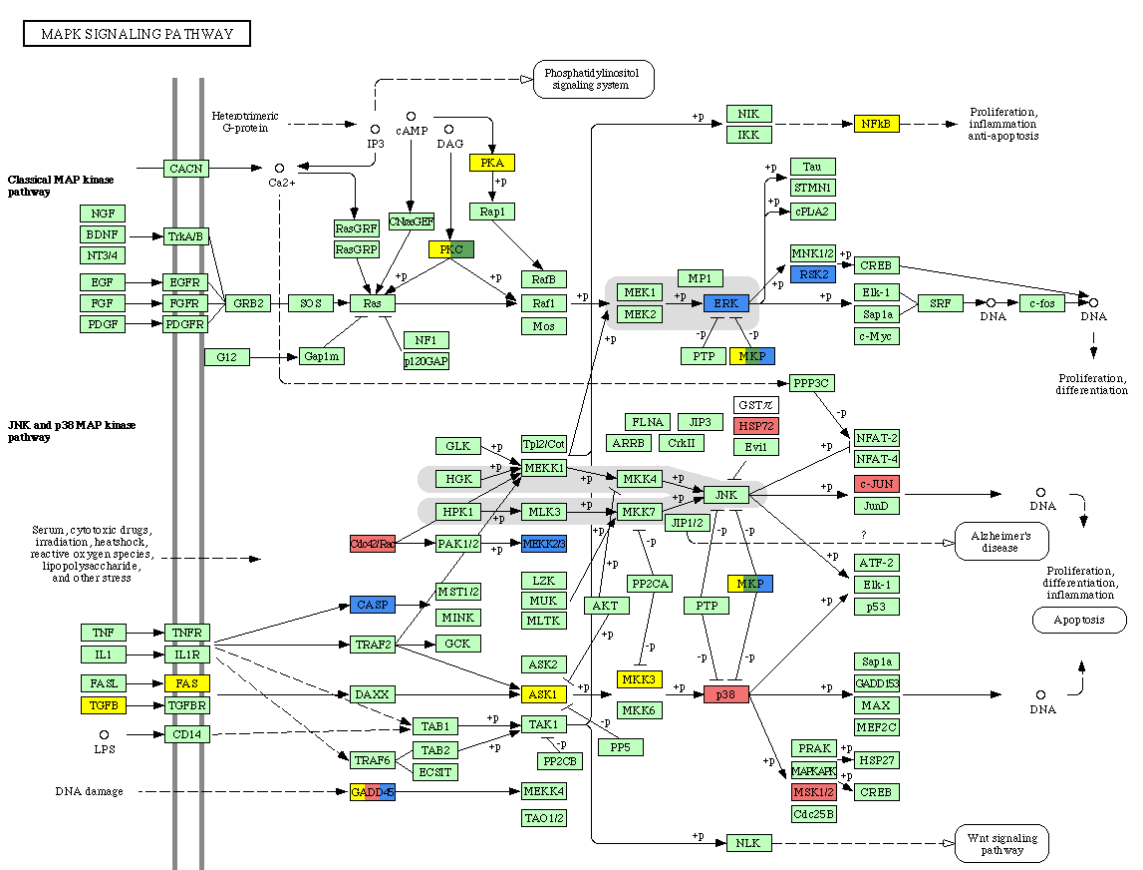
MAP kinase pathway analysis of the *meta-modes*. Yellow boxes correspond to genes mapped to the *apoptosis meta-mode*, red boxes to *regulatory sequences *and blue to *signal transduction meta-mode*, respectively. Solid arrows indicate direct and dashed arrows indirect activation. (Detailed legend information can be found on the KEGG website [63])

Within the *meta-mode Signal transduction *four *sub-modes*, 3.2, 6.2, 12.2 and 13.2 were combined together. The MAP-kinase pathway (Figure 1) could be identified as the major signal transduction pathway in *sub-modes *3.2 and 12.2. *Sub-mode *6.2 encompassed the functions signal transduction and cell communication. The remaining *sub-mode *13.2 could not be mapped to a defined pathway, but the majority of genes within this *sub-mode *are associated with innate immunity and defense functions. Among these we identified relevant genes, also related to signal transduction, like CD86, BLNK. The transcription factors LMO2 and FLI1 were unique in *sub-mode *13.2 whereas MMP9, CD36, CTSK, C1QR1 and MYCL1 as a TF were also present in several other *sub-modes*.

**Figure 2:**
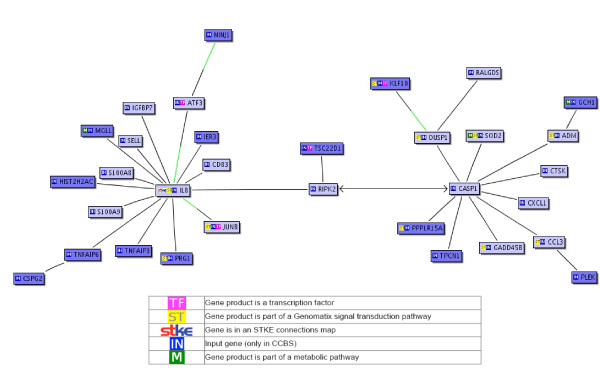
BiblioSphere pathway view shows the mapped Genes of *sub-mode *12.2. Genes passed the MAPK filter are highlighted blue. Cited relationships between two genes make up the edges. Display of edges is restricted to those that constitute the shortest path from the central node. If a gene that codes for a transcription factor is connected to a gene that is known to contain a binding site for this transcription factor in its promoter, the connecting line is colored green over half of its length near the gene containing the binding site. Arrowheads at the ends of a connecting line symbolize that gene X regulates gene Y.

The 12 and 18 respectively, identified MAPK-pathway genes were all unique within their *sub-modes *(Table 1), except IL8 and DUSP1, which were present in both *sub-modes*. IL8 is a member of the CXC chemokine family and thus one of the major mediators of the inflammatory response. It is also a potent angiogenic factor and has a signalling function in the FAS-pathway, whereas DUSP1 is assumed to play an important role in the human cellular response to environmental stress, as well as in the negative regulation of cellular proliferation. Another central gene of the MAPK-pathway is caspase-1 (CASP1), which was represented in *sub-mode *12.2 (Figure 2). Caspase-1 is responsible for the maturation of the multi-functional cytokine interleukin-1*β *and as member of the FAS caspase cascade it is involved in FAS mediated cell death [43]. Further remarkable genes associated with MAP-kinase in this *sub-mode *were S100A8, S100A9, GADD45B, CTSK, SOD2 and the transcription factors JUNB and ATF3, since they were all represented in other *sub-modes *or pathways, or play a central role in the MAPK-pathway.

**Table 1:** The table shows a comparison of the known M-CSF dependent macrophage differentiation pathways and the results of our gene expression mode analysis as described in the text. MM = meta-mode, PW = pathway, II & D = Innate immunity and defense, Cell C. = Cell communication, FAM = Fatty acid metabolism, DNA-P = DNA-protection.

**Known M-CSF dependent differentiation pathways**		**Meta-mode (MeSH Terms)**	**Pathway**	**Sub-mode**	**Mapped genes**		
					
					Total	MM	PW
Differentiation	PI3Akt JAK/STAT MEK/ERK	Signal transduction	MAPK	3.2	67	32	12
				12.2	53	40	18
			
			II & D	13.2	60	26	-
			
			Cell C.	6.2	62	29	29
		
	TP53 DNA protection	Regulatory sequences	JUN/FOS	4.1	43	22	3
				10.1	48	22	10
			
			FAM	11.2	47	23	12
			
			TP53 (DNA-P.)	14.1	64	34	12
		
	NF-*κ*B	Differentiation cell cycle	TP53 (DNA-protection)	5.2	43	25	8
				11.1	55	22	13
				12.1	64	23	12
		
Apoptosis		Survival Apoptosis	TP53 (DNA-protection)	2.1	55	17	5
				3.1	57	18	6
				6.1	74	28	12
				8.1	37	10	7
				9.2	43	19	8
				13.1	38	23	7
			
Survival	Prenylated-proteins Rho kinase		BAX	3.1	57	18	10
				8.1	37	10	11
				13.1	38	23	16
			
			CALR	4.2	58	24	6
			
			FAS	9.2	43	19	11

*Sub-mode *3.2 combined the MAPK-pathway with the thioredoxin (TRX) reductase/thioredoxin system. TRX is involved in a variety of oxidation reduction reactions that regulate cell growth and survival decisions [44]. It reduces ligand binding and DNA interaction by oxidizing cysteine residues within the DNA binding domain of glucocorticoid hormone receptors. Furthermore, TXNDC14 and TXNRD1 were found in this *sub-mode*. TRX also seems to be up-regulated by NGF through MAPK1 [45]. Other genes associated with the MAPK-pathway were: STK17A, SH3BP5, RPS6KA1, CD44, G6PD, IL1RN and the transcription factors EGR2.

In *sub-mode *6.2 all of the 29 genes involved in signal transduction were also related to the MeSH-term *cell communication*. Five of those signalling genes CFLAR, TXNDC1, YWHAZ, NOTCH2 and PSEN1 were also involved in the negative regulation of cell death.

### Regulatory Sequences

The MeSH-term *regulatory sequences *is described as nucleic acid sequences involved in gene expression regulation. This *meta-mode *combines genes mapped to the TP53-pathway (*sub-mode *14.1) and genes related to the oncogenes JUN/FOS (sub-mode 4.1 and 10.1), which are members of a family of transcription factors containing the basic-region-leucine zipper or bZIP motif. The BiblioSphere software did not define a specific pathway for *sub-mode *11.2, but there were a couple of peptidases and proteinases like LYZ, GGH and CPM as well as a remarkable number of classical targets for the SREBP transcription factors, regulating cholesterol and fatty acid metabolism: SQLE, CYP51A1, HMGCR, FDFT1, INSIG1, IDI1, SC5DL and LDLR.

*Sub-mode *14.1 represented an intersection of genes involved in gene expression regulation and the TP53 pathway. Genes which fulfill both criteria were ADM, CCND2, CD59, CDC42, DUSP6, GADD45A, GCH1, IER3, NDUFV2, PIM1, SLC2A3 and UBE3A. Moreover, *sub-mode *14.1 received high significance values (Z-Score) for the three other *meta-mode *categories and was also the *sub-mode *with the highest amount of genes represented in other *sub-modes *as well. This can be interpreted as an evidence for the complex and networked nature of gene expression regulation and the interactivity of cellular pathways.

The transcription factor JUN also known as c-Jun belongs to the family of c-Jun N-terminal kinases (JNKs) which are important for development and survival of macrophages [46]. *Sub-modes *4.1 and 10.1 combined twelve genes with a known relationship to the JUN/FOS pathway: CCND2, CREM, CXCL1, GADD45A, IL1RN, JUN, MAPK13, MARCKS, RALA, PLAU, S100A8 and SOD2.

### Differentiation, Cell Cycle

The *meta-mode cell cycle *was completely governed by the TP53 pathway. Although all three *sub-modes *5.2, 11.1 and 12.1 represented TP53 related genes, the intersection of genes was marginal. Only the genes DUSP6, PCNA and PRKCA were mapped to the TP53 pathway and were also present in the *sub-modes *5.2 and 12.1. *Sub-mode *11.1 represented genes specialized in cell cycle pathways regulating the interphase and in particular the G1 phase, since it contained the genes PPP1R15A, DUT, CD44, CDKN1A and SMC4L1. *Sub-modes *5.2 and 12.1 mainly represented genes involved in cell growth and proliferation.

*Sub-mode *5.2 was characterized by the TP53 related genes DHFR, VCAN, APP, EIF2AK2 and the transcription factor HMGB2 and HMGB3. Here, the latter has not been mapped to TP53 pathway but is mentioned here because of its strong relation to HMGB2.

The unique TP53 genes in *sub-mode *12.1 were: CAMK1, CTSB, GSTN1, NME1, HMGCR, GSN, CYP51A1 and IL1RN.

### Survival/Apoptosis

Apoptosis related pathways play a major role during the differentiation of monocytes to macrophages. Here we introduce the term "*survival/apoptosis*" for the MeSH term apoptosis, because the identified apoptosis pathways here function as survival mechanisms for the differentiating cells. It has been shown, that an absence of M-CSF induces apoptosis in cultivated monocytes [47]. Since apoptosis is regulated through many different pathways and regulatory mechanisms, we could identify seven *sub-modes *(2.1, 3.1, 6.1, 8.1, 9.2, 13.1, 4.2) related to apoptosis. These could be classified to four different pathways involved in the regulation of apoptosis: TP53 pathway, BAX pathway, FAS-pathway and calreticulin (CALR) regulated apoptosis. Three of these *sub-modes *represented only one pathway. *Sub-modes *2.1, 6.1 were mapped to the TP53 pathway and *sub-mode *4.2 is governed by CALR regulated apoptosis, whereas the others could be mapped to more than one pathway.

Due to the strongly networked nature of biological regulatory mechanisms, a lot of genes involved in more than one pathway can be regarded as connections between those. Toshiyuki and Reed [48] showed that the human BAX-gene is directly regulated by TP53 (TP53), whereas BAX is participating in the regulation of endoplasmatic reticulum Ca^2+ ^[49] as well. In this way it acts as a gateway for selected apoptotic signals. This was represented by the *sub-modes *3.1, 8.1 and 13.1 which could comparably be mapped to the TP53 and BAX pathway. Sub-mode 8.1 here combined the most interesting combination of genes. The genes CCL3, CCND3, PAICS, FYB, AKAB1, IL1RN, CXCL1, MT1A and the TFs EGR2 and ATF3 could be implicated with BAX. These genes overlapped with five of the seven genes mapped to the TP53 pathway: ATF3, BAX, CSPG2, EIF5B and IL1RN. Furthermore, the metallo-thioneins which are suggested to regulate DNA binding activity of TP53, MT1A, MT1F, MT1B and MT1X were represented in this *sub-mode *[50].

The role of CALR as a major Ca^2+^-binding (storage) protein in the lumen of the endoplasmatic reticulum is well known [51]. Consequently, one might imagine that CALR is involved in the regulation of apoptotic signals. The following genes of *sub-mode *4.2 are related to CALR: SLC11A1, CD93, PROCR, NME1 and ATP2B1. All of these genes, except ATP2B1, passed the MeSH-filter apoptosis. The link to the TP53 pathway is the transcription factor FOXO1A (also found in *sub-mode *6.1) and PRKCB1, which is also involved in various other cellular signaling pathways.

The member of the TNF-receptor superfamily FAS plays a central role in the regulation of programmed cell death. *Sub-mode *9.2 contained eleven genes related to FAS: GSTM1, RALGDS, ALOX5, VCAN, S100A9, S100A8, VIL2, LY75, STAB1, HEBP2 and CD44.

### Otherwise Classified

Although not all *sub-modes *could be mapped to specific *meta-modes*, the remaining *sub-modes *still provide useful information. While the genes sorted to *sub-modes *7.1 and 7.2 deliver no significant pathway information, they share common behavior. Genes of *sub-mode *7.1 were all down-regulated in macrophages or up-regulated in monocytes, respectively, whereas genes of *sub-mode *7.2 were up-regulated in macrophages. Among these, known marker-genes for the different cell types could be identified: MNDA, FCN1 and the S100 calcium binding proteins S100A8, S100A9 and S100A12 as monocyte and IGF2R, TSPAN4, MMP9, CTSK, MMD, TNS1 and CALR as macrophage genes.

Furthermore, the *sub-modes *5.1, 4.1, 8.2 and 14.2 contained Major Histocompatibility Class (MHC) genes. Whereas the *sub-mode *5.1 genes HLA-A and HLA-C belong to MHC class I, the MHC genes of the three other *sub-modes *belong to MHC class II which are: HLA-DQB1, HLA-DQA1, HLA-DPB1, HLA-DPA1 and HLA-DMB.

Additional meta-mode tables are shown in Additional file 1.

## Conclusion

It has been stated [52,53] that the use of ICA for the analysis of gene expression data is a promising tool, but there is still a lack of a careful discussion of the results. Here we emphasized the exploration of the biological relevance and obtained a detailed insight into the networked structure of the underlying regulatory mechanisms. Two MAP kinase related pathways could be identified as the main regulatory pathways during differentiation: the classical MAP kinase pathway and the JNK and p38 MAP kinase pathway, see figure 1. These results confirm expectations, according to which the MAP kinase pathway is activated by the M-CSF stimulus and functions as the main signal transduction pathway triggering macrophage differentiation and related pathways.

The conspicuous presence of TP53 associated pathways in M-CSF induced monocyte differentiation is associated with a dramatic regulation of cell-cycle and apoptosis related genes. This leads to the assumption that human mononuclear phagocytes, which are considered to be arrested to non-proliferating cells, still preserve proliferative potential [54].

Furthermore, we could show that ICA is able to distinguish between monocytes and macrophages concerning differential gene expression. This helpful attribute can be used to find specific marker genes not only for different cell types as it is shown here, but also for different tissues or normal and tumor cells.

**Figure 3:**
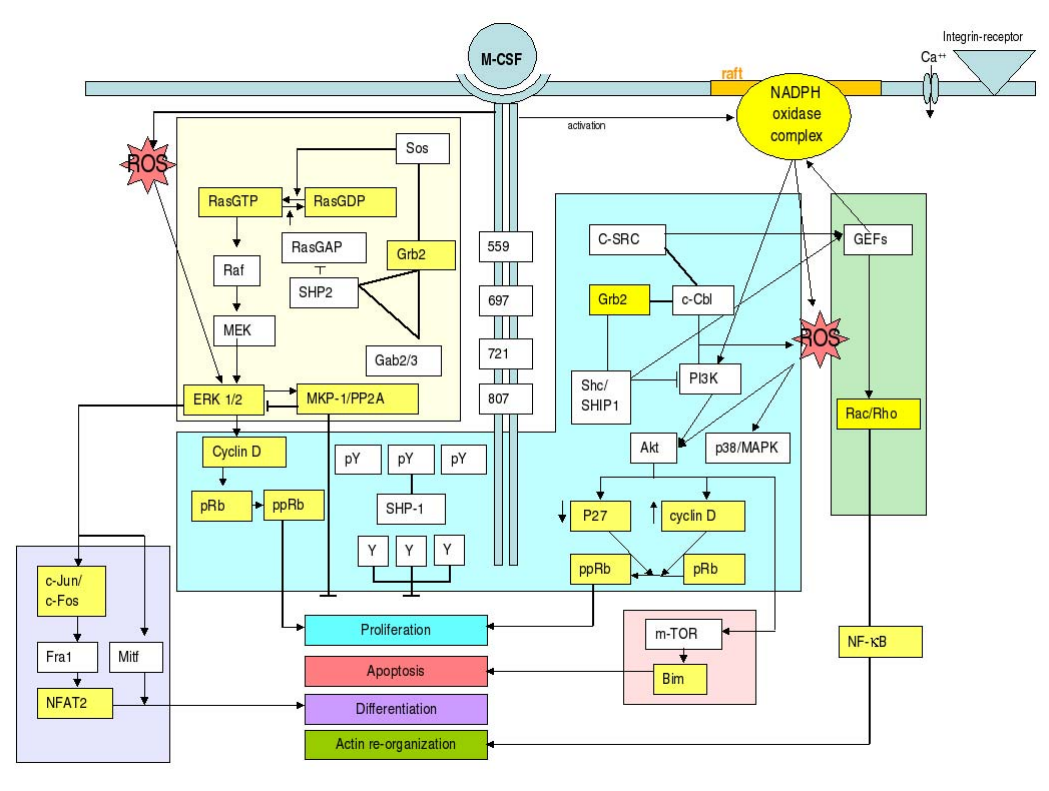
Overview over the main pathways involved in M-CSF dependent differentiation. The blue, purple, green and red colored boxes mediate proliferation, differentiation, actin re-organization and apoptosis. The yellow box mediates those common to proliferation and differentiation. Genes or gene products colored in intense yellow could be identified with our ICA analysis.

Moreover, we were able to identify different regulatory mechanisms during M-CSF dependent differentiation. Although signal transduction pathways are mainly regulated by protein modifications like phosphorylation or acetylation, genes associated to specific pathways involved in macrophage differentiation could be separated into *sub-modes *only by analyzing gene expression signatures and their related gene expression modes. Furthermore, this analysis could be improved by combining gene expression *sub-modes *extracted from different microarray experiments into informative gene expression *meta-modes*. The results are in full agreement with the experimental literature on M-CSF dependent differentiation [55] and illustrate the potential power of such information-theory-based, unsupervised and data-driven analysis methods, see Figure 3.

To fully explore the potential of such information-theory-based unsupervised analysis tools and especially to determine the suitability and reliability of ICA for the analysis of microarray datasets, further investigations are needed. The algorithms still suffer from the fact, that the number of estimated independent components, i.e. the extracted gene expression modes, depends on the number of available gene expression signatures and the dimension of the related gene expression profiles. Therefor, the availability of greater datasets should lead to advancements, and as shown here, greater datasets can be obtained by the careful combination of smaller datasets.

## Methods

### Dataset

For our analysis we combined the gene-chip results from three different experimental settings. In each experiment human peripheral blood monocytes were isolated from healthy donors (experiment 1 and 2) and from donors with Niemann-Pick type C disease (experiment 3). Monocytes were differentiated to macrophages for 4 days in the presence of M-CSF (50 ng/ml, R&D Systems). Differentiation was confirmed by phase contrast microscopy. Gene expression profiles were determined using Affymetrix HG-U133A (experiment 1 and 2) and HG-U133plus2.0 (experiment 3) GeneChips covering 22215 probe sets and about 18400 transcripts (HG-U133A). Probe sets only covered by HG-U133plus2.0 array were excluded from further analysis. In experiment one pooled RNA was used for hybridization, while in experiment two and tree RNA from single donors were used. The final data set consisted of seven monocyte and seven macrophage expression profiles and contained 22215 probe sets. After filtering out probe sets which had at least one absent call, 5969 probe sets remained for further analysis. The complete data set is publicly available in the NCBI Gene Expression Omnibus [56] through the accession number GSE9801.

### Preprocessing

**Figure 4:**
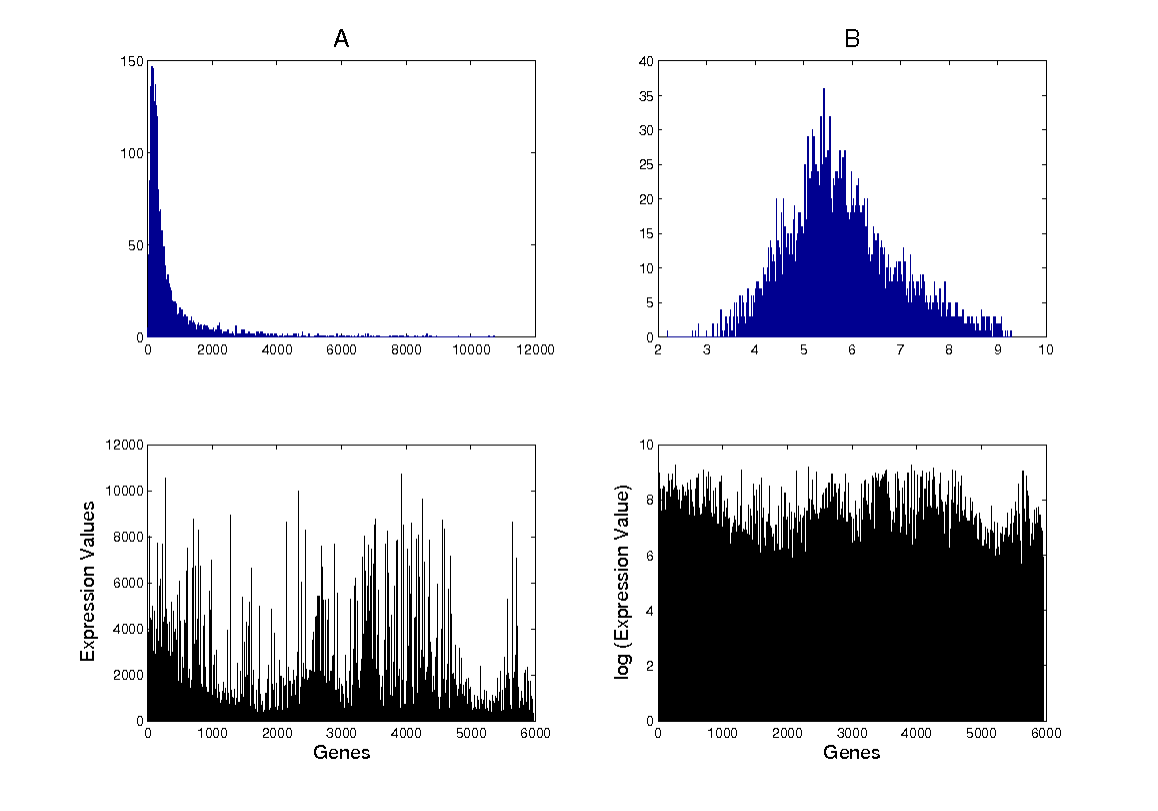
Histograms and expression profiles of an untransformed (A) and logarithmic corrected (B) microarray expression data set.

The bulk of preprocessing has been done using the Affymetrix GeneChip Operating Software (GCOS), where default presets were used. Additionally, we applied a logarithmic correction to the data. This has been done because effects with multiplicative behavior, which may contain biological relevant information, become linear after logarithmic transformation. Another reason is that, untransformed microarray expression profiles have a strongly skewed, hence unbalanced distribution. This means, that there is a large amount of expression values near zero whereas only very few genes show high expression levels (Figure 4). To avoid adverse effects caused by such unbalanced distributions, we applied a logarithmic transformation. The final data are usually represented as a data matrix whose columns represent expression signatures of N genes while the rows represent M corresponding gene expression profiles.

### JADE-based extraction of gene expression modes

The *Joint Approximative Diagonalization of Eigenmatrices (JADE) *algorithm has been proposed by Cardoso and Souloumiac [57,58]. It is a nearly exact algebraic approach to perform ICA.

The algorithm JADE is based on fourth-order *cumulant tensors ***T**_*z *_of pre-whitened input data **z **= **Qx **given by

(1) $$\begin{array}{lll} Cum(z_{i},z_{j},z_{k},z_{l}) & = & E\{z_{i}z_{j}z_{k}z_{l}\}-E\{z_{i}z_{j}\}E\{z_{k}z_{l}\}\\ & - & E\{z_{i}z_{k}\}E\{z_{l}z_{j}\}\\ & - & E\{z_{i}z_{l}\}E\{z_{j}z_{k}\} \end{array}$$

with the kurtosis $\kappa_{i}^{\left(4\right)}$ = *Cum*(*z*_*i*_*z*_*i*_*z*_*i*_*z*_*i*_) being the corresponding autocumulant. Associated with these cumulants is a fourth-order signal space (FOSS) which defines the range of all mappings *T*_*z *_: **M **→ **T**_*z*_(**M**)

(2) $$m_{ij}\to {\left[T_{z}(M)\right]}_{ij}=\sum_{k,l=0}^{m-1}Cum(z_{i},z_{j},z_{k},z_{l})M_{kl}$$

The corresponding matrices [**T**_*z *_(**M**)]_*ij *_will be called *cumulant matrices *in the following. Note that the dimensionality *m *of the FOSS equals at most the number of sources.

A spectral representation of the cumulant matrices can be obtained using the column vectors of the whitened mixing matrix with the corresponding eigenvalues related to the kurtosis of the independent components. This spectral representation can be used to obtain an eigenmatrix decomposition of the cumulant tensor according to

(3) **T**_*z*_(**E**^(*q*)^) = *μ*_*q*_**E**^(*q*)^

with 0 ≤ *q *≤ *m*^2 ^symmetric eigenmatrices **E**^(*q*) ^= **u**_*q*_$u_{q}^{T}$ and **u**_*q *_the q-th column of the mixing matrix **U**, and *μ*_*q *_being a scalar eigenvalue.

After whitening, a *m *× *m *– dimensional orthogonal matrix **D **= [**d**^(0) ^... **d**^(*m*-1)^], which jointly diagonalizes all eigenmatrices of **T**_*z*_, is found by maximizing the *joint diagonality criterion*

(4) $$c(D)=\sum_{q=0}^{m^{2}-1}{\left|Diag\left(D^{T}E^{\left(q\right)}D\right)\right|}^{2}$$

where *Diag*(·) denotes the vector of diagonal matrix elements. The joint diagonalizer **D **is then equivalent to the whitened mixing matrix **U**, hence the unknown independent component expression mode can be estimated easily.

### Sub-modes and meta-modes

As result of an ICA analysis of a set of gene expression signatures representing the rows of the transpose data matrix **X**^*T*^, we obtain a matrix **S **of independent components (the rows of **S**) which represent independent gene expression modes (GEMs) as well as a matrix **A **of basis vectors of the new feature space. To deduce meaningful biological information from the GEMs, the discovery of specific biological processes, which determine the modes, is the goal of our expression mode analysis. After decomposing the data matrix with ICA, each GEM has been split into two *sub-modes *which can be considered to feature genes which are co-expressed, thus co-regulated by the underlying regulatory process. A GEM consists of scores of gene contributions to the *sub-modes *which account for the observation that excitatory as well as inhibitory regulations exist. In order to extract the most significant genes, various statistical tools can be applied which, however, often suffer from the small M large N case. Therefore, in most cases a threshold is simply applied, or, after ranking, a fixed number of top and bottom genes are chosen and further analyzed [59]. The rational behind these methods is that each extracted gene expression *sub-mode *is best represented by its most active genes. However, the choice of threshold or number of active genes is non-trivial, and will influence the results considerably. In this study we assume instead that mapping to distinct pathways is most non-ambiguous by using a relatively small number of genes.

**Figure 5:**
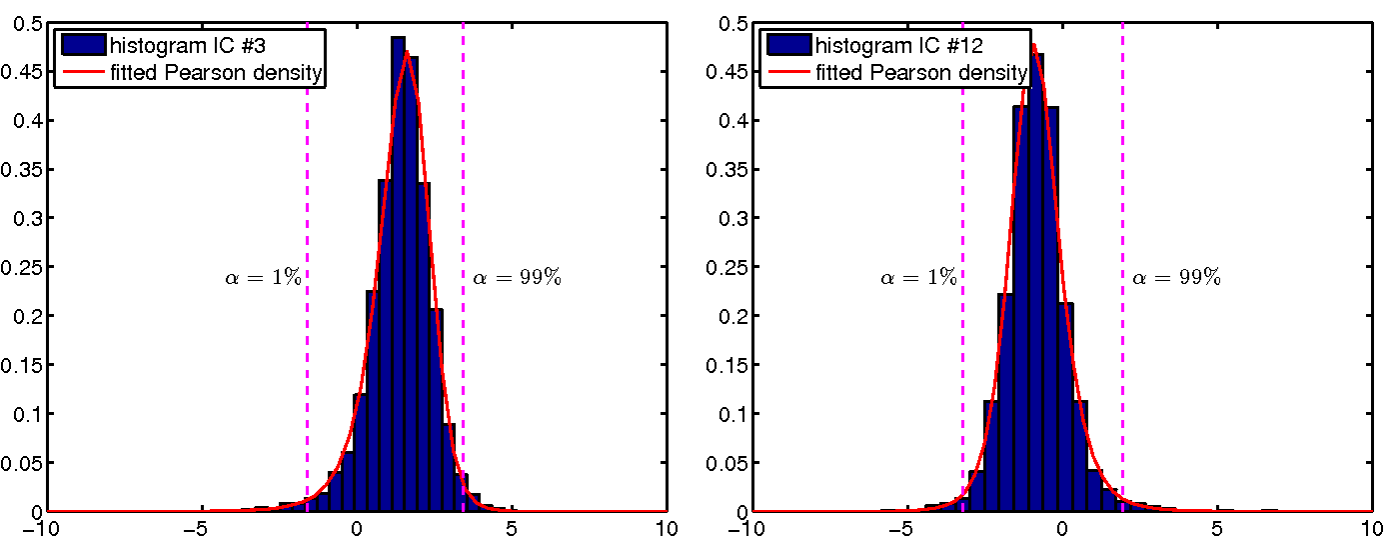
Maximum-likelihood Pearson fit of the EM-densities, for EM number 3 in (a) and number 12 in (b). The corresponding four moments are *μ*(*y*_3_) = 1.4, *σ*(*y*_3_) = 1.0, skewness(*y*_3_) = -0.95 and kurtosis(*y*_3_) = 4.0 for (a) and *μ*(*y*_3_) = -0.84, *σ*(*y*_3_) = 1.0, skewness(*y*_3_) = 0.49 and kurtosis(*y*_3_) = 4.4 for (b).

Here, we took a different approach by selecting genes that are extremal with respect to some probabilistic model. For each GEM *y*(*i*) ∈ ℝ, where *i *indexes the genes, we calculated the first four central moments corresponding to mean, standard deviation, skewness and kurtosis of the underlying data distribution. These shape parameters are then used to fit a density according to the Pearson family [60] using maximum-likelihood, see Figure 5. We chose a Pearson density as prior since it allows for flexible modeling with respect to these first four moments, which seemed crucial as for example skewness varies considerably between modes, see Figure 5, and high kurtotic as well as close-to-Gaussian modes were present.

We then used the estimated Pearson densities to determine the 1 - *α *and *α *percentiles for *α *= 1%. Samples that lie below the 1-percentile are denoted as significantly down-regulated genes, and genes above the 99-percentile as significantly up-regulated genes. The corresponding *sub-modes *were labeled as *i*.1 for down-regulation and *i*.2 for up-regulation. In Table 2 we list the number of significant genes in each *sub-mode*.

**Table 2:** Number of selected down- and up-regulated genes in each gene expression mode (GEM).

EM	*n*_down_	*n*_up_
1	68	112
2	59	59
3	69	79
4	54	64
5	74	47
6	88	65
7	54	68
8	43	64
9	59	51
10	51	38
11	64	59
12	71	62
13	43	73
14	79	34

### Mode analysis

We analyzed the gene *sub-modes *with BiblioSphere [61]. BiblioSphere is a data mining tool intended to provide gene relationships from literature databases and genome-wide promoter analysis. The probe sets were mapped to transcripts and to known genes with use of the Genomatix database. To uncover the biological meaning of the genes in the *sub-mode*, we applied the MeSH-Filter (Medical Subject Headings) to our data, which is the National Library of Medicine's controlled vocabulary thesaurus. We decided to use the category *biological sciences *as filter criterion. Co-citations between the genes of the *sub-mode *were taken into account by using the literature mining tool of the BiblioSphere software. Interesting terms were identified through Z-Scores which indicate over-representation of genes in the referring biological categories. Z-Scores are given by *Z - Score *= (*n *- $\hat{n}$)/*σ*_*n *_where *n *is the number of observed genes meeting any given criterion, $\hat{n}$ is the corresponding expected number and *σ*_*n *_gives the standard deviation of *n*. All terms mentioned in this work are significant with respect to the Genomatix guidelines.

Depending on our filter analysis we defined several *meta-modes*, where we combined *sub-modes *with similar categories. In some cases we subclassified *sub-modes *within one *meta-mode*. In this way 4 meta-modes could be generated, whereas 17 of 28 *sub-modes *could be mapped to at least one *meta-mode*. For some *meta-modes *we displaced the MeSH-Term category with additional categories with respect to the underlying biology.

Additionally we used the KEGG pathway database for biochemical pathway analysis to more thoroughly characterize the biological relevance of a *meta-mode*. The genes corresponding to the *meta-modes *were mapped on database pathways using Pathway-Express which is part of the Onto-Tools provided by Intelligent Systems and Bioinformatics Laboratory [62].

## Authors' contributions

DL conceived the study, performed bioinformatical analysis and drafted the manuscript. PU helped with the biochemical analysis. MG and EO performed the microarray studies. FT and EWL guided the bioinformatical analysis. GS guided the study and coordinated the project.

## Supplementary Material

Additional file 1 **Meta-modes tables**. Additional tables are provided in the pdf document. Click here for file
