# Supplementary material for: Analyzing M-CSF dependent monocyte/macrophage differentiation: Expression modes and meta-modes derived from an independent component analysis
Source: BMC Bioinformatics. 2008 Feb 17;9:100. doi: 10.1186/1471-2105-9-100 (PMC2277398; doi:10.1186/1471-2105-9-100)
Supplement: Additional file 1 — Meta-modes tables. Additional tables are provided in the pdf document. [file 1471-2105-9-100-S1.pdf]

Table 3 - Signal transduction genes

SYMBOL	NAME	Pathway	CLU	ProbeSet
ADAM10	ADAM metallopeptidase domain 10	Cell C.	6.2	214895_S_AT
ADM	adrenomedullin	MAPK	12.2	202912_AT
AIF1	allograft inflammatory factor 1		13.2	209901_X_AT
ALDH1A1	aldehyde dehydrogenase 1 family, member A1		12.2	212224_AT
ARF1	ADP-ribosylation factor 1	Cell C.	6.2	208750_S_AT
ARFGEF1	ADP-ribosylation factor guanine nucleotide-exchange factor 1(brefeldin A-inhibited)		6.2	216266_S_AT
ATF3	activating transcription factor 3	MAPK	12.2	202672_S_AT
BCL3	B-cell CLL/lymphoma 3		3.2	204908_S_AT
BID	BH3 interacting domain death agonist		3.2	204493_AT
BIRC1	baculoviral IAP repeat-containing 1		13.2	204860_S_AT
BLNK	B-cell linker		13.2	207655_S_AT
C1QR1	complement component 1, q subcomponent, receptor 1		13.2	202878_S_AT
CAMKK2	calcium/calmodulin-dependent protein kinase kinase 2, beta		3.2	212252_AT
CASP1	caspase 1, apoptosis-related cysteine peptidase (interleukin 1, beta, convertase)	MAPK	12.2	211368_S_AT
CCL3	chemokine (C-C motif) ligand 3	MAPK	12.2	205114_S_AT
CD163	CD163 antigen		13.2	215049_X_AT
CD36	CD36 antigen (collagen type I receptor, thrombospondin receptor)		13.2	206488_S_AT
CD44	CD44 antigen (homing function and Indian blood group system)	MAPK	3.2	217523_AT
CD58	CD58 antigen, (lymphocyte function-associated antigen 3)		6.2	216942_S_AT
CD83	CD83 antigen (activated B lymphocytes, immunoglobulin superfamily)	MAPK	12.2	204440_AT
CD86	CD86 antigen (CD28 antigen ligand 2, B7-2 antigen)		13.2	205686_S_AT
CFLAR	CASP8 and FADD-like apoptosis regulator	Cell C.	6.2	211317_S_AT
CSPG2	chondroitin sulfate proteoglycan 2 (versican)		12.2	221731_X_AT
CTSK	cathepsin K (pycnodysostosis)	MAPK	12.2,13.2	202450_S_AT
CXCL1	chemokine (C-X-C motif) ligand 1 (melanoma growth stimulating activity, alpha)	MAPK	12.2	209774_X_AT
CYP1B1	cytochrome P450, family 1, subfamily B, polypeptide 1		3.2,6.2	202435_S_AT
DDX3X	DEAD (Asp-Glu-Ala-Asp) box polypeptide 3, X-linked		6.2	212514_X_AT
DUSP1	dual specificity phosphatase 1	MAPK	12.2	201041_S_AT
EGR2	early growth response 2 (Krox-20 homolog, Drosophila)	MAPK	3.2	205249_AT
EREG	epiregulin		3.2	205767_AT
FABP5	fatty acid binding protein 5 (psoriasis-associated)		13.2	202345_S_AT
FCGR1A	Fc fragment of IgG, high affinity Ia, receptor (CD64)		13.2	214511_X_AT
FLI1	Friend leukemia virus integration 1		13.2	204236_AT
G6PD	glucose-6-phosphate dehydrogenase	MAPK	3.2	202275_AT
GADD45B	growth arrest and DNA-damage-inducible, beta	MAPK	12.2	209305_S_AT
GDI2	GDP dissociation inhibitor 2	Cell C.	6.2	200008_S_AT
H2BFS	H2B histone family, member S		3.2	209806_AT
HOMER3	homer homolog 3 (Drosophila)	Cell C.	6.2	215489_X_AT
HPSE	heparanase		13.2	219403_S_AT
IER3	immediate early response 3		12.2	201631_S_AT
IFITM3	interferon induced transmembrane protein 3 (1-8U)		12.2	201315_X_AT
IGFBP7	insulin-like growth factor binding protein 7	MAPK	12.2	201163_S_AT
IL1RN	interleukin 1 receptor antagonist	MAPK	3.2	212657_S_AT
IL2RG	interleukin 2 receptor, gamma (severe combined immunodeficiency)		3.2	204116_AT
IL8	interleukin 8	MAPK	3.2,12.2	202859_X_AT
JUNB	jun B proto-oncogene	MAPK	12.2	201473_AT
KLF10	Kruppel-like factor 10		3.2,12.2	202393_S_AT
LMO2	LIM domain only 2 (rhombotin-like 1)		13.2	204249_S_AT
M6PR	mannose-6-phosphate receptor (cation dependent)		6.2	200900_S_AT
MAP3K2	mitogen-activated protein kinase kinase kinase 2	Cell C.	6.2	221695_S_AT
MAPK1	mitogen-activated protein kinase 1	MAPK	3.2	208351_S_AT
MCP	membrane cofactor protein (CD46, trophoblast-lymphocyte cross-reactive antigen)		6.2	207549_X_AT
MGLL	monoglyceride lipase		12.2	211026_S_AT
MMP9	matrix metallopeptidase 9 (gelatinase B, 92kDa gelatinase, 92kDa type IV collagenase)		13.2	203936_S_AT
MTSS1	metastasis suppressor 1		13.2	203037_S_AT
MYCL1	v-myc myelocytomatosis viral oncogene homolog 1, lung carcinoma derived (avian)		13.2	214058_AT

continued on next page

continued from previous page

SYMBOL	NAME	Pathway	CLU	ProbeSet
NEK3	NIMA (never in mitosis gene a)-related kinase 3		13.2	211089_S_AT
NFKBIE	nuclear factor of kappa light polypeptide gene enhancer in B-cells inhibitor, epsilon		3.2	203927_AT
OGT	O-linked N-acetylglucosamine (GlcNAc) transferase	Cell C.	6.2	207564_X_AT
PDE4B	phosphodiesterase 4B, cAMP-specific (phosphodiesterase E4 dunce homolog, Drosophila)		12.2	203708_AT
PECAM1	platelet/endothelial cell adhesion molecule (CD31 antigen)		13.2	208981_AT
PLEK	pleckstrin		12.2	203471_S_AT
PPP1R15A	protein phosphatase 1, regulatory (inhibitor) subunit 15A		12.2	37028_AT
PRNP	prion protein (p27-30)		6.2	215707_S_AT
PSEN1	presenilin 1 (Alzheimer disease 3)	Cell C.	6.2	207782_S_AT
PTGER2	prostaglandin E receptor 2 (subtype EP2), 53kDa		12.2	206631_AT
PTPRO	protein tyrosine phosphatase, receptor type, O		13.2	208121_S_AT
RALGDS	ral guanine nucleotide dissociation stimulator	MAPK	12.2	209050_S_AT
RIPK2	receptor-interacting serine-threonine kinase 2	MAPK	12.2	209545_S_AT
RPS6KA1	ribosomal protein S6 kinase, 90kDa, polypeptide 1	MAPK	3.2	203379_AT
S100A8	S100 calcium binding protein A8 (calgranulin A)	MAPK	12.2	202917_S_AT
S100A9	S100 calcium binding protein A9 (calgranulin B)	MAPK	12.2	203535_AT
SCAMP1	secretory carrier membrane protein 1		6.2	206668_S_AT
SCAP2	src family associated phosphoprotein 2	Cell C.	6.2	216899_S_AT
SELL	selectin L (lymphocyte adhesion molecule 1)	MAPK	12.2	204563_AT
SEPT2	septin 2		6.2	200778_S_AT
SH3BP5	SH3-domain binding protein 5 (BTK-associated)	MAPK	3.2	201811_X_AT
SLA	Src-like-adaptor		13.2	203761_AT
SLC3A2	solute carrier family 3 (activators of dibasic and neutral amino acid transport), member 2		3.2	200924_S_AT
SNAP23	synaptosomal-associated protein, 23kDa		6.2	214544_S_AT
SOD2	superoxide dismutase 2, mitochondrial	MAPK	12.2	215223_S_AT
STK17A	serine/threonine kinase 17a (apoptosis-inducing)	MAPK	3.2	202693_S_AT
TALDO1	transaldolase 1		3.2	201463_S_AT
TLK1	tousled-like kinase 1		3.2	202606_S_AT
TLR4	toll-like receptor 4	Cell C.	6.2,13.2	221060_S_AT
TNFAIP3	tumor necrosis factor, alpha-induced protein 3		3.2, 12.2	202644_S_AT
TNFAIP6	tumor necrosis factor, alpha-induced protein 6		12.2	206026_S_AT
TPP1	tripeptidyl peptidase I		6.2	214196_S_AT
TSC22D1	TSC22 domain family, member 1		12.2	215111_S_AT
TXN	thioredoxin	MAPK	3.2	208864_S_AT
TXNDC	thioredoxin domain containing	Cell C.	6.2	208097_S_AT
TXNIP	thioredoxin interacting protein		6.2,13.2	201008_S_AT
TXNRD1	thioredoxin reductase 1		3.2	201266_AT
UCP2	uncoupling protein 2 (mitochondrial, proton carrier)		13.2	208997_S_AT
VAMP3	vesicle-associated membrane protein 3 (cellubrevin)		6.2	201337_S_AT
YWHAZ	tyrosine 3-monooxygenase/tryptophan 5-monooxygenase activation protein, zeta polypeptide		6.2	200641_S_AT
ZNFN1A1	zinc finger protein, subfamily 1A, 1 (Ikaros)		6.2	205039_S_AT

Table 4 - Regulatory sequences genes

SYMBOL	NAME	Pathway	CLU	ProbeSet
ABCA1	ATP-binding cassette, sub-family A (ABC1), member 1		4.1	203505_AT
ABCG1	ATP-binding cassette, sub-family G (WHITE), member 1		10.1	204567_S_AT
ACAT2	acetyl-Coenzyme A acetyltransferase 2 (acetoacetyl Coenzyme A thiolase)		11.2	209608_S_AT
ADM	adrenomedullin	TP53	14.1	202912_AT
ALDH1A1	aldehyde dehydrogenase 1 family, member A1		4.1,11.2	212224_AT
ALDH2	aldehyde dehydrogenase 2 family (mitochondrial)		4.1	201425_AT
ALOX5AP	arachidonate 5-lipoxygenase-activating protein		10.1	204174_AT
ARTS-1	type 1 tumor necrosis factor receptor shedding aminopeptidase regulator		11.2	210385_S_AT
C3AR1	complement component 3a receptor 1		4.1, 14.1	209906_AT
CALR	calreticulin		10.1	214315_X_AT
CCND2	cyclin D2	JUN/FOS,TP53	10.1, 14.1	200953_S_AT
CDC42	cell division cycle 42 (GTP binding protein, 25kDa)	TP53	14.1	208727_S_AT
CPM	carboxypeptidase M		11.2	206100_AT
CREM	cAMP responsive element modulator	JUN/FOS	10.1	207630_S_AT
CTSK	cathepsin K (pycnodysostosis)		4.1	202450_S_AT
CTSL	cathepsin L		14.1	202087_S_AT
CXCL1	chemokine (C-X-C motif) ligand 1 (melanoma growth stimulating activity, alpha)	JUN/FOS	10.1,14.1	209774_X_AT
CXCR4	chemokine (C-X-C motif) receptor 4		4.1	211919_S_AT
CYP51A1	cytochrome P450, family 51, subfamily A, polypeptide 1		11.2	216607_S_AT
EBP	emopamil binding protein (sterol isomerase)		11.2	202735_AT
FBP1	fructose-1,6-bisphosphatase 1		10.1	209696_AT
FDFT1	farnesyl-diphosphate farnesyltransferase 1		11.2	208647_AT
FYB	FYN binding protein (FYB-120/130)		4.1	211795_S_AT
G0S2	G0/G1switch 2		14.1	213524_S_AT
G1P2	interferon, alpha-inducible protein (clone IFI-15K)		4.1	205483_S_AT
GADD45A	growth arrest and DNA-damage-inducible, alpha	JUN/FOS, TP53	10.1, 14.1	203725_AT
GCH1	GTP cyclohydrolase 1 (dopa-responsive dystonia)	TP53	14.1	204224_S_AT
GGH	gamma-glutamyl hydrolase (conjugase, folylpolygammaglutamyl hydrolase)		11.2	203560_AT
GM2A	GM2 ganglioside activator		4.1	212737_AT
HLA-DMB	major histocompatibility complex, class II, DM beta		4.1	203932_AT
HLA-DQA2	major histocompatibility complex, class II, DQ alpha 2		4.1, 14.1	212671_S_AT
HLA-DQB1	major histocompatibility complex, class II, DQ beta 1		4.1	212998_X_AT
HMGCR	3-hydroxy-3-methylglutaryl-Coenzyme A reductase		11.2	202540_S_AT
HPSE	heparanase		14.1	219403_S_AT
HSPA1B	heat shock 70kDa protein 1B		11.2	200800_S_AT
IER3	immediate early response 3	TP53	14.1	201631_S_AT
IL1RN	interleukin 1 receptor antagonist	JUN/FOS	10.1	212659_S_AT
INSIG1	insulin induced gene 1		11.2	201625_S_AT
JUN	v-jun sarcoma virus 17 oncogene homolog (avian)	JUN/FOS	4.1,10.1	201466_S_AT
LCP2	lymphocyte cytosolic protein 2 (SH2 domain containing leukocyte protein of 76kDa)		14.1	205269_AT
LDLR	low density lipoprotein receptor (familial hypercholesterolemia)		11.2	202068_S_AT
LOC440607	Fc-gamma receptor I B2		10.1	214511_X_AT
LYZ	lysozyme (renal amyloidosis)		11.2, 14.1	213975_S_AT
MAPK13	mitogen-activated protein kinase 13	JUN/FOS	10.1	210058_AT
MARCKS	myristoylated alanine-rich protein kinase C substrate	JUN/FOS	4.1, 14.1	201670_S_AT
MMP14	matrix metallopeptidase 14 (membrane-inserted)		10.1	160020_AT
NISCH	nischarin		4.1	201591_S_AT
NP	nucleoside phosphorylase		14.1	201695_S_AT
PDGFC	platelet derived growth factor C		4.1	218718_AT
PFKFB3	6-phosphofructo-2-kinase/fructose-2,6-biphosphatase 3		14.1	202464_S_AT
PHLDA1	pleckstrin homology-like domain, family A, member 1		14.1	217996_AT
PIM1	pim-1 oncogene	JUN/FOS, TP53	10.1, 14.1	209193_AT
PLAU	plasminogen activator, urokinase	JUN/FOS	10.1	211668_S_AT
PROCR	protein C receptor, endothelial (EPCR)		14.1	203650_AT
PTGS1	prostaglandin-endoperoxide synthase 1 (prostaglandin G/H synthase and cyclooxygenase)		11.2	215813_S_AT
RALA	v-ral simian leukemia viral oncogene homolog A (ras related)		10.1	214435_X_AT
RDX	radixin		4.1	212397_AT
RPS6KA4	ribosomal protein S6 kinase, 90kDa, polypeptide 4		11.2	204632_AT
S100A12	S100 calcium binding protein A12 (calgranulin C)		14.1	205863_AT

continued on next page

continued from previous page

SYMBOL	NAME	Pathway	CLU	ProbeSet
S100A8	S100 calcium binding protein A8 (calgranulin A)	JUN/FOS	10.1	202917_S_AT
SHMT2	serine hydroxymethyltransferase 2 (mitochondrial)		10.1	214437_S_AT
SLC11A1	solute carrier family 11 (proton-coupled divalent metal ion transporters), member 1		10.1	210423_S_AT
SOD2	superoxide dismutase 2, mitochondrial	JUN/FOS	4.1	215223_S_AT
SPINT2	serine peptidase inhibitor, Kunitz type, 2		14.1	210715_S_AT
SQLE	squalene epoxidase		11.2	209218_AT
TNFAIP6	tumor necrosis factor, alpha-induced protein 6		14.1	206026_S_AT
TRAPPC2	trafficking protein particle complex 2		4.1	209751_S_AT
TRIB3	tribbles homolog 3 (Drosophila)		14.1	218145_AT
UGCG	UDP-glucose ceramide glucosyltransferase		4.1,10.1	204881_S_AT

Table 5 - Differentiation and cell cycle genes

SYMBOL	NAME	Pathway	CLU	ProbeSet
AGA	aspartylglucosaminidase	TP53	11.1	204333_S_AT
ALCAM	activated leukocyte cell adhesion molecule		12.1	201951_AT
ALOX5	arachidonate 5-lipoxygenase		11.1	204446_S_AT
APP	amyloid beta (A4) precursor protein (peptidase nexin-II, Alzheimer disease)	TP53	5.2	214953_S_AT
ATP1B1	ATPase, Na+/K+ transporting, beta 1 polypeptide		12.1	201242_S_AT
CD44	CD44 antigen (homing function and Indian blood group system)	TP53	11.1	210916_S_AT
CDKN1A	cyclin-dependent kinase inhibitor 1A (p21, Cip1)	TP53	11.1	202284_S_AT
CSPG2	chondroitin sulfate proteoglycan 2 (versican)	TP53	5.2	221731_X_AT
CTNNB1	catenin (cadherin-associated protein), beta 1, 88kDa		11.1	201533_AT
CYP51A1	cytochrome P450, family 51, subfamily A, polypeptide 1	TP53	12.1	216607_S_AT
DUSP6	dual specificity phosphatase 6	TP53	5.2,12.1	208892_S_AT
DUT	dUTP pyrophosphatase		5.2,11.1	209932_S_AT
EIF2AK2	eukaryotic translation initiation factor 2-alpha kinase 2	TP53	5.2	204211_X_AT
EPRS	glutamyl-prolyl-tRNA synthetase		12.1	200842_S_AT
EREG	epiregulin		11.1	205767_AT
F8	coagulation factor VIII, procoagulant component (hemophilia A)		5.2	205756_S_AT
FCGR1A	Fc fragment of IgG, high affinity Ia, receptor (CD64)		5.2	216950_S_AT
FCGR3A	Fc fragment of IgG, low affinity IIIa, receptor (CD16a)		5.2	204007_AT
FYN	FYN oncogene related to SRC, FGR, YES		5.2	210105_S_AT
GCLC	glutamate-cysteine ligase, catalytic subunit		12.1	202923_S_AT
GGH	gamma-glutamyl hydrolase (conjugase, folylpolygammaglutamyl hydrolase)		5.2, 12.1	203560_AT
GSN	gelsolin (amyloidosis, Finnish type)	TP53	12.1	200696_S_AT
HMGB2	high-mobility group box 2	TP53	5.2	208808_S_AT
HMGB3	high-mobility group box 3		5.2, 11.1	203744_AT
HMGCR	3-hydroxy-3-methylglutaryl-Coenzyme A reductase	TP53	12.1	202540_S_AT
IL1RN	interleukin 1 receptor antagonist	TP53	12.1	212659_S_AT
ITGA4	integrin, alpha 4 (antigen CD49D, alpha 4 subunit of VLA-4 receptor)		5.2	205885_S_AT
LDLR	low density lipoprotein receptor (familial hypercholesterolemia)		5.2	202068_S_AT
LMNB1	lamin B1		5.2	203276_AT
LYZ	lysozyme (renal amyloidosis)		5.2	213975_S_AT
MCM5	MCM5 minichromosome maintenance deficient 5, cell division cycle 46 (S. cerevisiae)		5.2	216237_S_AT
NME1	non-metastatic cells 1, protein (NM23A) expressed in	TP53	12.1	201577_AT
PCNA	proliferating cell nuclear antigen	TP53	5.2,12.1	201202_AT
PDCD4	programmed cell death 4 (neoplastic transformation inhibitor)		5.2	212593_S_AT
PICALM	phosphatidylinositol binding clathrin assembly protein		11.1	212511_AT
PPP1R15A	protein phosphatase 1, regulatory (inhibitor) subunit 15A	TP53	11.1	37028_AT
PRKCA	protein kinase C, alpha	TP53	5.2	213093_AT
RRM1	ribonucleotide reductase M1 polypeptide		5.2	201477_S_AT
RUNX3	runt-related transcription factor 3	TP53	11.1	204198_S_AT
SELL	selectin L (lymphocyte adhesion molecule 1)		5.2	204563_AT
SLA	Src-like-adaptor		12.1	203761_AT
SLC7A1	solute carrier family 7 (cationic amino acid transporter, y+ system), member 1		12.1	212295_S_AT
SMARCA3	SWI/SNF related, matrix associated, actin dependent regulator of chromatin, subfamily a, member 3	TP53	5.2	202983_AT
SMC4L1	SMC4 structural maintenance of chromosomes 4-like 1 (yeast)		11.1	201664_AT
SOX4	SRY (sex determining region Y)-box 4		11.1	201417_AT
SPTBN1	spectrin, beta, non-erythrocytic 1		5.2	212071_S_AT
SRD5A1	steroid-5-alpha-reductase, alpha polypeptide 1 (3-oxo-5 alpha-steroid delta 4-dehydrogenase alpha 1)	TP53	11.1	204675_AT
TFDP1	transcription factor Dp-1		5.2	212330_AT

Table 6 - Survival/Apoptosis genes

SYMBOL	NAME	Pathway	CLU	ProbeSet
ADAM17	ADAM metallopeptidase domain 17 (tumor necrosis factor, alpha, converting enzyme)	TP53, BAX	13.1	205746_S_AT
ALOX5	arachidonate 5-lipoxygenase	BAX, FAS	3.1, 9.2	204446_S_AT
ALOX5AP	arachidonate 5-lipoxygenase-activating protein	BAX	3.1	204174_AT
ATF3	activating transcription factor 3	TP53, BAX	8.1	202672_S_AT
BAX	BCL2-associated X protein	TP53	3.1, 8.1,13.1	211833_S_AT
BCL2A1	BCL2-related protein A1		4.2	205681_AT
BTG1	B-cell translocation gene 1, anti-proliferative		13.1	200920_S_AT
C1QR1	complement component 1, q subcomponent, receptor 1	CALR	4.2	202878_S_AT
CACYBP	calcyclin binding protein		2.1	210691_S_AT
CALR	calreticulin		4.2	214315_X_AT
CCL3	chemokine (C-C motif) ligand 3	BAX	6.1 8.1	205114_S_AT
CCND2	cyclin D2		4.2	200953_S_AT
CD36	CD36 antigen (collagen type I receptor, thrombospondin receptor)	CALR	4.2	209555_S_AT
CD44	CD44 antigen (homing function and Indian blood group system)	TP53, FAS	9.2	204490_S_AT
CD83	CD83 antigen (activated B lymphocytes, immunoglobulin superfamily)	BAX	13.1	204440_AT
CHMP5	chromatin modifying protein 5		2.1	219356_S_AT
CSPG2	chondroitin sulfate proteoglycan 2 (versican)	TP53, FAS	9.2	221731_X_AT
CTSD	cathepsin D (lysosomal aspartyl peptidase)	TP53, BAX	3.1	200766_AT
CXCL1	chemokine (C-X-C motif) ligand 1 (melanoma growth stimulating activity, alpha)	BAX	6.1 8.1	204470_AT
CXCR4	chemokine (C-X-C motif) receptor 4	BAX	3.1,13.1	217028_AT
CYP51A1	cytochrome P450, family 51, subfamily A, polypeptide 1	TP53	2.1	202314_AT
DNM2	dynamin 2		4.2	202253_S_AT
DNTTIP2	deoxynucleotidyltransferase, terminal, interacting protein 2		6.1	202776_AT
DUSP1	dual specificity phosphatase 1	TP53, BAX	13.1	201041_S_AT
EGR2	early growth response 2 (Krox-20 homolog, Drosophila)	BAX	8.1,13.1	205249_AT
EIF5B	eukaryotic translation initiation factor 5B	TP53	8.1	201027_S_AT
ERCC1	excision repair cross-complementing rodent repair deficiency, complementation group 1		4.2	203719_AT
F8	coagulation factor VIII, procoagulant component (hemophilia A)		9.2	205756_S_AT
FAS	Fas (TNF receptor superfamily, member 6)	TP53	9.2	204780_S_AT
FCGR1A	Fc fragment of IgG, high affinity Ia, receptor (CD64)		3.1	216950_S_AT
FLJ22386	leucine zipper domain protein		13.1	218394_AT
FOXO1A	forkhead box O1A (rhabdomyosarcoma)	BAX	4.2, 6.1, 13.1	202724_S_AT
FYB	FYN binding protein (FYB-120/130)	BAX	8.1	211795_S_AT
FYN	FYN oncogene related to SRC, FGR, YES	BAX	13.1	210105_S_AT
GADD45A	growth arrest and DNA-damage-inducible, alpha	TP53, BAX	3.1, 6.1	203725_AT
GRB10	growth factor receptor-bound protein 10		9.2	209409_AT
HEBP2	heme binding protein 2	FAS	9.2	203430_AT
HLA-DQA1	major histocompatibility complex, class II, DQ alpha 1	TP53	2.1, 3.1, 13.1	213831_AT
IER3	immediate early response 3	P53	6.1	201631_S_AT
IGFBP7	insulin-like growth factor binding protein 7		2.1	201163_S_AT
IL1RN	interleukin 1 receptor antagonist	TP53, BAX	8.1,13.1	212659_S_AT
ING1	inhibitor of growth family, member 1	TP53, BAX	13.1	208415_X_AT
IRS2	insulin receptor substrate 2	BAX	13.1	209185_S_AT
ITGAL	integrin, alpha L (antigen CD11A (p180), lymphocyte function-associated antigen 1; alpha polypeptide)	BAX	13.1	213475_S_AT
JAG1	jagged 1 (Alagille syndrome)	P53	6.1	209099_X_AT
LAMP1	lysosomal-associated membrane protein 1	BAX	3.1	201551_S_AT
LNK	lymphocyte adaptor protein		4.2	203320_AT
LRMP	lymphoid-restricted membrane protein		3.1	35974_AT
LY75	lymphocyte antigen 75	FAS	9.2	205668_AT
MAP2K3	mitogen-activated protein kinase kinase 3		4.2	215498_S_AT
MAP3K5	mitogen-activated protein kinase kinase kinase 5		6.1	203836_S_AT
MCL1	myeloid cell leukemia sequence 1 (BCL2-related)		4.2	200798_X_AT
NDUFA5	NADH dehydrogenase (ubiquinone) 1 alpha subcomplex, 5, 13kDa	TP53	2.1	201304_AT
NEDD8	neural precursor cell expressed, developmentally down-regulated 8		2.1	201840_AT
NFKB2	nuclear factor of kappa light polypeptide gene enhancer in B-cells 2 (p49/p100)	TP53, BAX	13.1	207535_S_AT
NME1	non-metastatic cells 1, protein (NM23A) expressed in	CALR	4.2	201577_AT
OLR1	oxidised low density lipoprotein (lectin-like) receptor 1		13.1	210004_AT
PCBP2	poly(rC) binding protein 2		13.1	213263_S_AT

continued on next page

continued from previous page

SYMBOL	NAME	Pathway	CLU	ProbeSet
PCNA	proliferating cell nuclear antigen	TP53	2.1	201202_AT
PDE4B	phosphodiesterase 4B, cAMP-specific (phosphodiesterase E4 dunce homolog, Drosophila)		13.1	203708_AT
PER2	period homolog 2 (Drosophila)		6.1	205251_AT
PLEK	pleckstrin		13.1	203470_S_AT
PPP1R15A	protein phosphatase 1, regulatory (inhibitor) subunit 15A	TP53, BAX	13.1	37028_AT
PRKACB	protein kinase, cAMP-dependent, catalytic, beta		3.1	202741_AT
PRKCB1	protein kinase C, beta 1		4.2	209685_S_AT
PROCR	protein C receptor, endothelial (EPCR)	CALR	4.2	203650_AT
PSMB8	proteasome (prosome, macropain) subunit, beta type, 8 (large multifunctional peptidase 7)		9.2	209040_S_AT
RAD23B	RAD23 homolog B (S. cerevisiae)		2.1	201222_S_AT
RALGDS	ral guanine nucleotide dissociation stimulator	FAS	9.2	209050_S_AT
REL	v-rel reticuloendotheliosis viral oncogene homolog (avian)	BAX	13.1	206036_S_AT
RNASE2	ribonuclease, RNase A family, 2 (liver, eosinophil-derived neurotoxin)		9.2	206111_AT
RSL1D1	ribosomal L1 domain containing 1		2.1, 4.2	212018_S_AT
S100A8	S100 calcium binding protein A8 (calgranulin A)	TP53, FAS	9.2	202917_S_AT
S100A9	S100 calcium binding protein A9 (calgranulin B)	TP53, FAS	9.2	203535_AT
SERBP1	SERPINE1 mRNA binding protein 1		9.2	210466_S_AT
SFRS5	splicing factor, arginine/serine-rich 5		13.1	212266_S_AT
SLC11A1	solute carrier family 11 (proton-coupled divalent metal ion transporters), member 1	CALR	4.2,9.2	210423_S_AT
SOD2	superoxide dismutase 2, mitochondrial	TP53	2.1	216841_S_AT
SOX4	SRY (sex determining region Y)-box 4		13.1	201417_AT
SPTBN1	spectrin, beta, non-erythrocytic 1		13.1	212071_S_AT
STEAP3	STEAP family member 3		9.2	218424_S_AT
SUB1	SUB1 homolog (S. cerevisiae)		2.1	214512_S_AT
TANK	TRAF family member-associated NFKB activator		2.1	209451_AT
TGFB1	transforming growth factor, beta 1 (Camurati-Engelmann disease)	TP53, BAX	13.1	203085_S_AT
TNFSF10	tumor necrosis factor (ligand) superfamily, member 10	TP53, BAX	2.1, 3.1, 6.1	202688_AT
TNFSF13	tumor necrosis factor (ligand) superfamily, member 13	BAX	3.1	210314_X_AT
TRIB3	tribbles homolog 3 (Drosophila)		4.2	218145_AT
UBE1C	ubiquitin-activating enzyme E1C (UBA3 homolog, yeast)		2.1	209115_AT
VIL2	villin 2 (ezrin)	TP53, FAS	9.2	208623_S_AT
WARS	tryptophanyl-tRNA synthetase		6.1	200629_AT
